# Expression of interleukin-1 (IL-1) ligands system in the most common endometriosis-associated ovarian cancer subtypes

**DOI:** 10.1186/1757-2215-3-3

**Published:** 2010-01-28

**Authors:** Mamadou Keita, Paul Bessette, Manuella Pelmus, Youssef Ainmelk, Aziz Aris

**Affiliations:** 1 Department of Obstetrics and Gynecology, Sherbrooke University Hospital Centre, 3001, 12e Avenue Nord, Sherbrooke, Quebec J1H 5N4, Canada; 2 Department of Pathology, Sherbrooke University Hospital Centre, 3001, 12e Avenue Nord, Sherbrooke, Quebec J1H 5N4, Canada; 3 Clinical Research Centre of Sherbrooke University Hospital Centre, 001, 12e Avenue Nord, Sherbrooke, Quebec J1H 5N4, Canada

## Abstract

**Objectives:**

Endometrioid carcinoma of the ovary is one of the most types of epithelial ovarian cancer associated to endometrioisis. Endometrioid tumors as well as endometriotic implants are characterized by the presence of epithelial cells, stromal cells, or a combination of booth, that resemble the endometrial cells, suggesting a possible endometrial origin of these tumors. Pro-inflammatory cytokines, including interleukin-1 (IL-1) have been reported to be involved in both endometriosis and ovarian carcinogenesis. The major objective of this study was to determine the level expression of IL-1 ligands system (IL-1α, IL-1β and IL-1RA) in the most common subtypes of ovarian cancer cells compared to endometrial cells.

**Methods:**

We used primary endometrial cells, endometrial cell line RL-952 and different subtypes of epithelial ovarian cancer cell lines including TOV-112D (endometrioid), TOV-21G (clear cell) and OV-90 (serous). Immunofluorescence and real-time PCR analysis were used respectively for detecting IL-1 ligands at the levels of cell-associated protein and mRNA. Soluble IL-1 ligands were analyzed by ELISA.

**Results:**

We demonstrated that IL-1 ligands were expressed by all endometriosis-associated ovarian cancer subtypes and endometrial cells. In contrast to other cancer ovarian cells, endometrioid cells exhibit a specific decrease of cell-associated IL-1RA expression and its soluble secretion.

**Conclusion:**

Endometrioid ovarian cancer exhibits an alteration in the expression of IL-1RA, a key protector against tumorogenic effects of IL-1. This alteration evokes the same alteration observed in endometriotic cells in previous studies. This suggests a possible link between the endometrium, the tissue ectopic endometriosis and endometrioid ovarian cancer.

## Background

Ovarian cancer, the leading cause of death from gynecological malignancy, is the seventh most common malignancy in women worldwide. In more than two thirds of the cases are diagnosed at advanced stages [1]. Ovarian cancer has been reported in patients with pre-existing endometriosis, known as endometriosis-associated ovarian cancer (EAOC) [2,3]. It has been reported an increased risk of ovarian cancer in women with endometriosis [2,3]. Endometriosis is a common benign disease defined by the presence of endometrial glands and stroma in ectopic locations, mainly ovary and peritoneum. Ovarian endometrioid cells resemble to endometrial cells, mimicking the structure of endometrium, is one of the most frequent histological subtypes of EAOC [2,3].

The menstrual phase of the endometrium and ovary includes inflammation as a physiologic component [4-9]. Thus IL-1, a major pro-inflammatory cytokine, is physiologically involved in the process of ovulation [10-14] and implantation [15,16]; and pathologically in epithelial ovarian carcinoma [17-21], endometrial tumors [9,22] and endometriosis [23]. Several experimental data support a crucial role of IL-1 as an autocrine and paracrine stimulus in murine and human carcinogenesis [24,25]. IL-1 potentates invasiveness and metastasis of malignant cells, by inducing adhesion molecule expression on tumor as well as on the endothelial cells [24-27]. Moreover, IL-1 increases the growth of ovarian carcinoma cells [28] and its proliferation [29].

IL-1 ligands system includes IL-1 alpha (IL-1α) and IL-1 beta (IL-1β) which are potent active cytokines, while IL-1 receptor antagonist (IL-1 RA) acts as an inhibitor cytokine. It may exert its effects in a soluble extracellular (sIL-1RA) and intracellular (icIL-1RA) forms [30,31]. IL-1 RA competes with IL-1α and IL-1β in binding to IL-1 receptors without inducing a cellular response [32].

Many studies have shown that the concentrations of IL-1β were significantly increased in peritoneal fluid [33], ectopic, and eutopic endometrial cells [34] from women with endometriosis, suggesting that IL-1β could induce the growth, adhesion [9], invasiveness [35], and angiogenesis [36] of endometrial fragments outside of the uterus. As a competitive antagonist for IL-1β, IL-1RA is detected in eutopic endometrium but is completely decreased in peritoneal fluid [37] or absent in ectopic endometrium [38] of patients with endometriosis. This suggests that an imbalance between the levels of IL-1β and its natural receptor antagonist may contribute to the unrestricted growth of ectopic endometrium. However, little is known about IL-1 ligands system expression in endometrioid ovarian cells, given the hypothesis that this tissue is of endometrial origin.

Since impairment of IL-1 activity regulation in ectopic cells may promote a neoplastic transformation in the ovary [9,39,40], we hypothesized that IL-1RA may play a role in the pathogenesis of endometriosis-associated ovarian cancer.

## Methods

### Cells, antibodies, and others reagents

Primary epithelial cells from the endometrium, well-differentiated endometrial carcinoma RL952 and immortalized malignant endometrioid ovarian cancer cell TOV-112D (EOCC), clear cell ovarian cancer cell TOV-21G, serous ovarian cancer cell OV-90 cell lines (ATCC, Rockville, MD, USA) were used. Ovarian cancer and primary endometrial cells were cultured in medium 199 and medium 105 mixtures (Invitrogen Life Technologies Inc., New York, NY). RL-952 was maintained in Dulbecco's modified Eagle's medium F-12 (GIBCO: Invitrogen, NY, USA). These media were supplemented with 10% FBS. Hanks Balanced Salt Solution containing trypsin 0.25 mM EDTA was obtained from Sigma (St. Louis, MO, USA). The concentrations of human IL-1α, IL-1β and IL-1RA in cell culture supernatants were measured by using ELISA kit (R&D Systems Inc., Minneapolis, MN). Monoclonal mouse anti-human IL-1α and IL-1RA and antibody Alexa Fluor 594-labelled goat anti-mouse were respectively purchased from R&D Systems Inc. (Minneapolis, MN, USA) and Molecular Probes (Invitrogen, Carlsbad, CA, USA). 4, 6-diaminido-2-phenyl-indole (DAPI) was obtained from Sigma Aldrich (St Louis, MO, USA). Reverse Transcriptase Supercript II and SYBR Green Master Mix were purchased respectively from Invitrogen (Carlsbad, CA, USA) and Applied Biosystems (Foster City, CA, USA).

### Tissue dissociation and epithelial endometrial cells purification

Endometrial biopsies were obtained from 5 healthy fertile patients undergoing gynecological surgery for tubal ligation. The study was approved by the CHUS Ethics Human Research Committee on Clinical Research. All participants gave written consent. Tissues were washed in HBSS minced into small pieces and dissociated with collagenase as previously described [41]. Endometrium was finely minced and incubated in sterile Hank's balanced salt solution (HBSS) (GIBCO Invitrogen Corp., Burlington, ON, Canada) containing 20 mM Hepes, 100 U/ml penicillin, 100 μg/ml streptomycin and 1 mg/ml collagenase at 37°C in a shaking water bath during 60 minutes. Fragments of epithelial glands from collegenase digestion were isolated by filtration through a 45-μm nylon mesh.

### Enzyme-linked immunosorbent assay for IL-1β and IL-1RA proteins

Endometrial and ovarian cancer cells were seeded at a density of 2 × 10^6 ^cells per 1 ml in 12-well plates containing medium with 10% FBS and cultured overnight. Medium was exchanged and cells were cultured for a further 48 hr. The culture supernatants were collected and microfuged at 1,500 rpm for five min to remove particles and the supernatants frozen at -20°C until use in ELISA. The concentration of IL-1α, IL-1β and IL-1RA in the supernatants per 2 × 10^6 ^cells was measured using an ELISA kit (R&D Systems, Minneapolis, MN) according to the manufacturer's instructions.

### Immunofluorescence and quantitative imaging cytometry of IL-1α and IL-1RA proteins

To evaluate intracellular, membrane-bound IL-1α and intracellular IL-1RA, immunostaining was performed according to Akoum et al. [42]. Briefly, cell lines were grown on glass coverslips overnight and fixed with formaldehyde in PBS. The cells were permeabilized by treatment with 0.1% Triton X-100 (PBS/TX) in PBS for 15 min at room temperature and incubated with a monoclonal mouse anti-human IL-1α or IL-1RA antibody in 1% BSA/PBS for 2 hours. After washing with PBS, the cells were incubated with secondary antibody goat Anti-Mouse Alexa Fluor 594 for 1 hour. Nuclei were identified by 4', 6'-diamidino-2-phenylindole staining for 15 min at room temperature. Following mounting, cells were observed under the Leica microscope. Experiments have been done five times. Immunostained cells were scanned with iCys imaging cytometer (Compucyte, Cambridge, MA). Immuno-staining was detected using Argon ion (488 nm) excitation laser with green (530 nm/30 nm) detection PMT. DNA staining was detected using violet diode (405 nm) excitation laser with bleu (463 nm/39 nm) detection PMT. Image for cellular morphology was acquired using scattering of the Argon ion laser. Scanning was performed at 0,5 μm × 0,25 μm pixel size resolution. Cellular event selection was performed using a virtual channel obtained by adding green and blue fluorescence signals to insure detection and quantification of cytoplasmic signal. Immuno-staining intensity and cellular area were measured and used to compare IL-1α and IL-1RA proteins expression between EOCC, EC and the others subtypes of ovarian cancers. An experimented scorer selected the scoring thresholds for immuno-staining intensity. All cell selections were confirmed by visualizing a gallery of at least 250 representative cells.

### Real time PCR analysis of IL-1α, IL-1β and IL-1RA mRNA

IL-1α, IL-1β and IL-1RA mRNA extraction was achieved using trizol. To evaluate the level of gene expression, real-time PCR with SYBR Green dye was applied. Experiments have been done five times. The Rotor-Gene (Corbett Research, Sydney, Australia) equipment for reaction monitoring was used. β actine gene was used as internal control. The forward sequence GAATGACgCCCTCAATCAAAGT and reverse sequence TCATCTTGGGCAGTCACATACA were used for human IL-Rα. For human IL-1RA, the forward and reverse sequences were AATCCAGCAAGATGCAAGCC and ACGCCTTCGTCAGGCATATT, respectively. Forward and reverse sequences for human IL-1β were AAACAGATGAAGTGCTCCTTCCAGG and TGGAGAACACCACTTGTTGCTCCA respectively. For β actine, the forward and reverse sequences were CATGTACGTTGCTATCCAGGC and CTCCTTAATGTCACGCACGAT, respectively. The PCR reaction was performed in 20 μl final volume using 36-well plates. The reaction mixture contained 10 μl SyberGreenSuperMix, 100 nM of each primer (forward and reverse) and 1 μl cDNA. All samples were run in duplicate. The thermal protocol was as follows: 1 min 90°C, followed by 60 cycles (20 s at 95°C - denaturation, 20 s at 60°C - annealing and 20 s at 72°C - elongation - when the signal was acquired). Each sample was normalized on the basis of its GAPDH content according to the formula , EC representing endometrial cells; OCC, ovarian cancer cells and C_T _the threshold cycle.

### Statistical analysis

IL-1α and IL-1RA staining scores follow an ordinal scale. Data followed a parametric distribution and were shown as means ± SD. We used one-way analysis of variance (ANOVA) and the Bonferroni's test *post hoc *for multiple comparisons or the unpaired *t*-test for comparison of two groups. Statistical analyses were performed using excel and GraphPad Software, Prism 4.0 (GraphPad Software, San Diego, CA, USA). Differences were considered as statistically significant whenever a P value < 0.05 occurred.

## Results

Our results showed that IL-1α and IL-1RA were expressed in studied cells at levels of the protein, the mRNA and the soluble for. However, IL-1β was not detected inside cells at level of the protein.

### Immunofluorescence analysis of cellular IL-1α and IL-1RA proteins expression

**Figure 1:**
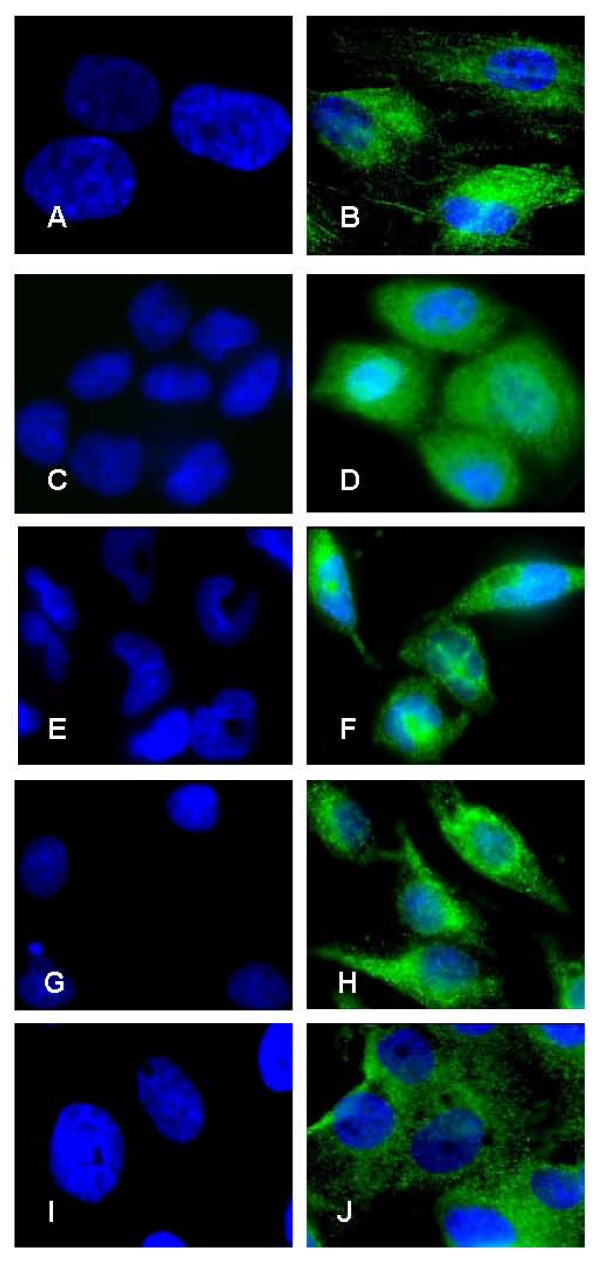
**Expression of IL-1α by immunofluorescence**. Expression of IL-1α protein in primary endometrial cells (A and B), endometrial cell line RL-952 (C and D) and the different subtypes of epithelial ovarian cancer cell lines TOV-112D (endometrioid), TOV-21G (clear cell) and OV-90 (serous) (E and F; G and H; I and J; respectively). Note the marked intensity of IL-1α staining in both endometrial cells (B and D) and ovarian cancer cells (F, H and J). No immunofluorescence was observed in negative controls for endometrial cells (A and C) and ovarian cancer cells (E, G, and I) in the absence of primary antibody (objective × 100).

**Figure 2:**
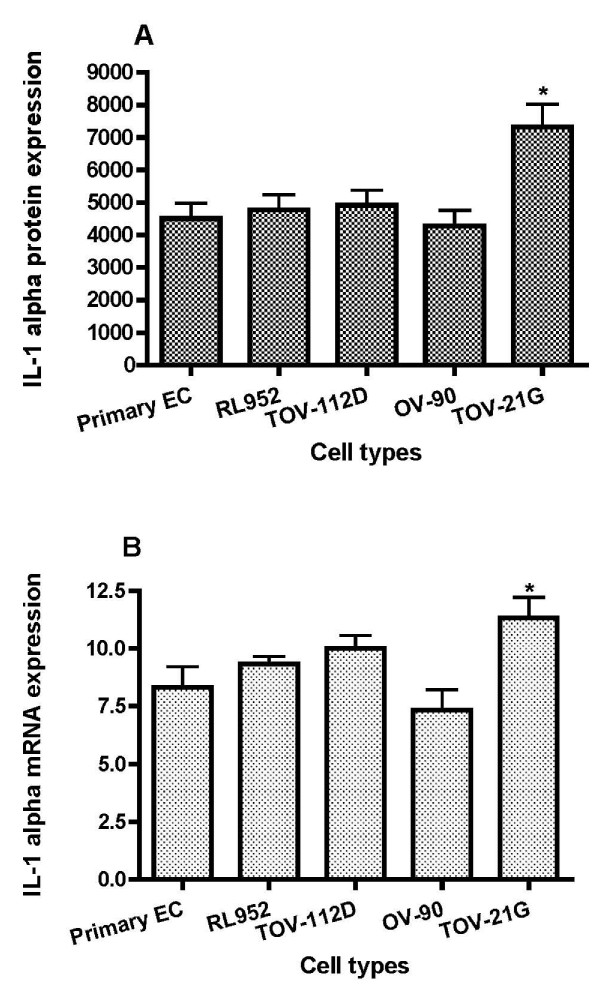
**Graphical illustration of IL-1α expression**. IL-1α expression scores in endometrial cells (EC) and epithelial ovarian cancer cells lines (mean ± SD). A: IL-1α was immunostained and immunofluorescence was scored using iCys imaging cytometer. B: expression of IL-1α in EC and epithelial ovarian cancer cells lines was detected by real time PCR using primers specific for IL-1α and β-actin.

**Figure 3:**
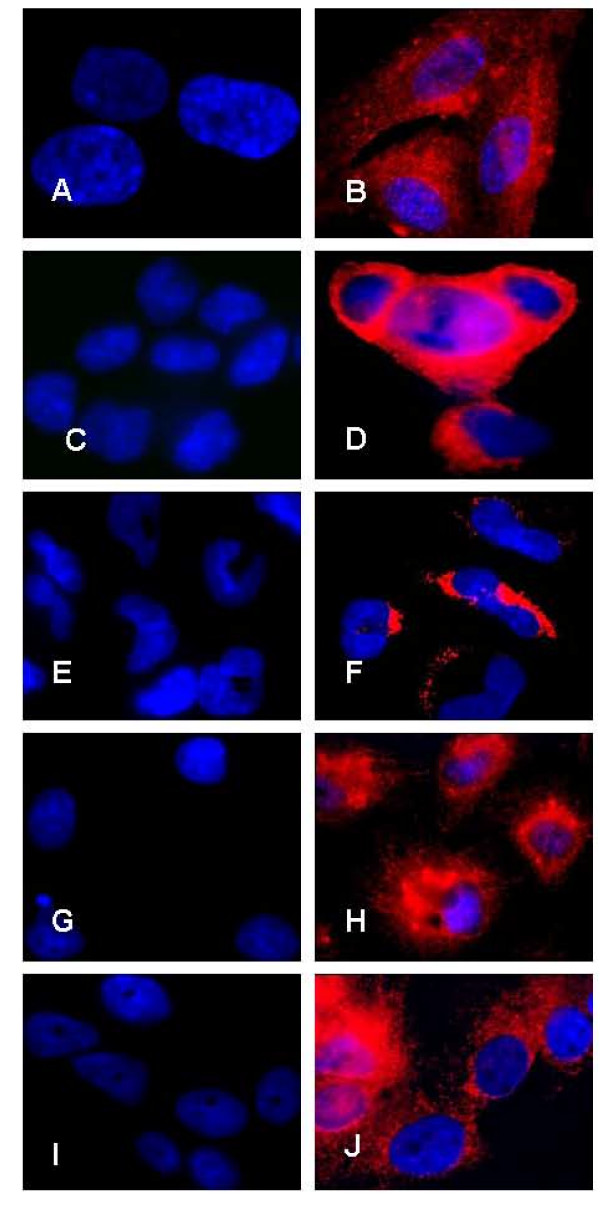
**IL-1RA expression by immunofluorescence**. Expression of IL-1RA protein in primary endometrial cells (A and B), endometrial cell line RL-952 (C and D) and the different subtypes of epithelial ovarian cancer cell lines TOV-112D (endometrioid), TOV-21G (clear cell) and OV-90 (serous) (E and F; G and H; I and J; respectively). Note the marked intensity of IL-1RA staining in both endometrial cells (B and D) and ovarian cancer cells (F, H and J). No immunofluorescence was observed in negative controls for endometrial cells (A and C) and ovarian cancer cells (E, G, and I) in the absence of primary antibody (objective × 100).

**Figure 4:**
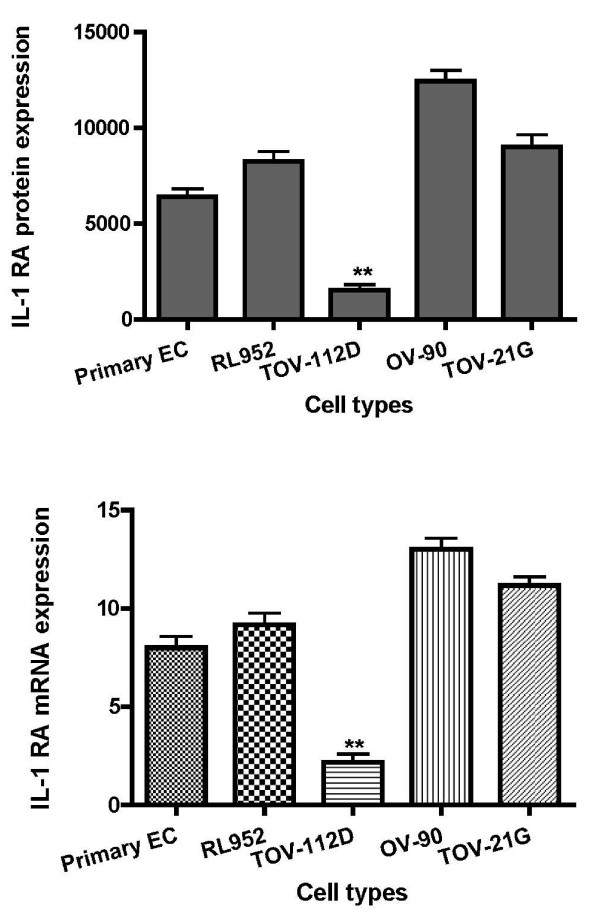
**Graphical illustration of IL-1RA expression**. IL-1RA expression scores in endometrial cells (EC) and epithelial ovarian cancer cells lines (mean ± SD). A: IL-1RA was immunostained and immunofluorescence was scored using iCys imaging cytometer. B: expression of IL-1RA in EC and epithelial ovarian cancer cells lines was detected by real time PCR using primers specific for IL-1RA and β-actin.

The intensity of IL-1 ligands system proteins staining was scored using quantitative imaging cytometry. Immunofluorescence analysis clearly showed that IL-1α protein (Figure 1 and Figure 2A) and IL-1RA protein (Figure 3 and Figure 4A) were expressed in all types of studied cells. Whereas incubation of cells without primary antibodies (negative controls), did not result in any noticeable staining. As shown in Table 1, statistical analysis comparing endometrial cells and EAOC subtypes showed that IL-1α staining was more intense in clear cell line (TOV-21G) (Figure 1 and Figure 2A; P < 0.05), whereas IL-1RA staining was higher in serous cell line (OV-90) and very low in endometrioid ovarian cell line (TOV-112D) (Figure 3 and Figure 4A; P < 0.05).

**Table 1:** Comparative expression of IL-1α, IL-1β and IL-1RA in endometrial cells and epithelial ovarian cancer cell lines

IL-1α (mean ± SD) n = 5	Protein (intensity)	ΔCt: mRNA	**2**^**-ΔΔCt**^
Primary EC (control)	4678.6 ± 473	14.3 ± 0.4	
RL-952	4948.3 ± 167	14.4 ± 0.6	1.1
TOV-112D	5217.1 ± 391	13.6 ± 0.1	1.3
TOV-21G	11320.1 ± 391*	13.2 ± 0.2	1.9*
OV-90	3897.6 ± 590	15 ± 0.6	0.8
**IL-1β (mean ± SD) n = 5**			

Primary EC (control)		11.4 ± 0.6	
RL-952		10.8 ± 0.4	1.3
TOV-112D		10.0 ± 0.3	2.0*
TOV-21G		10.7 ± 0.3	1.4
OV-90		9.9 ± 0.2	2.2*
**IL-1RA (mean ± SD) n = 5**			

Primary EC (control)	6921.1 ± 611	16.8 ± 0.7	
RL-952	8391.3 ± 241	15.9 ± 0.1	1.2
TOV-112D	2101.6 ± 352*	18.9 ± 0.6	0.2*
TOV-21G	8798.1 ± 571*	16.1 ± 0.4	1.3
OV-90	13251.6 ± 495	15.9 ± 0.3	1.4

**2^-ΔΔCt^**: fold differences of mRNA expression.

* P < 0.05, comparison to control (primary endometrial cells).

### Analysis of IL-1 ligands gene expression by Real Time PCR

**Figure 5:**
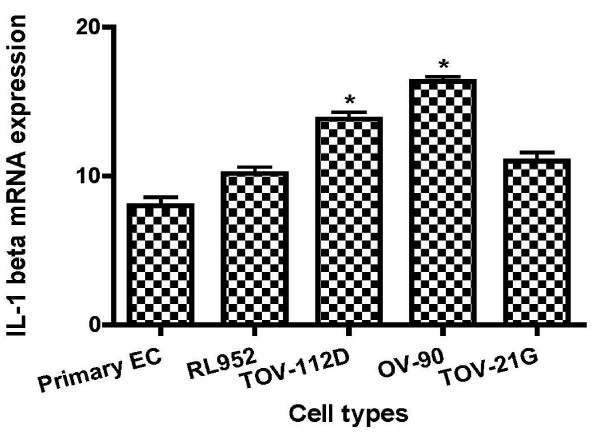
**Graphical illustration of IL-1β**. IL-1β gene expression in EC and epithelial ovarian cancer cells lines by real time PCR using primers specific for IL-1β and β actin.

To further analyze IL-1α and IL-1RA at level of transcription, gene expression was achieved by real-time quantitative PCR kinetics using SybrGreen I chemistry. The baseline adjustment method of the Rotor Gene software was used to determine the threshold cycle in each reaction. A melting curve was constructed for each primer pair to verify the presence of one gene-specific peak and the absence of primer dimmer. A representative Real-Time-PCR of IL-1α and IL-1RA mRNA in EAOC subtypes compared to endometrial cell line RL-952 and primary endometrial cells are shown in Table 1. IL-1α mRNA expression was higher in TOV-21G cells (Figure 2B; P < 0.05), whereas no statically changes of IL-1α mRNA expression was observed between endometrial cells and the other epithelial ovarian cancer cell lines (Figure 2B). Analysis of mRNA levels showed a marked decrease in the expression of IL-1RA in EOCC (Figure 4B; P < 0.001); and an increase in the expression of IL-1β in TOV-112D and OV-90 cells (Table 1, Figure 5).

### ELISA analysis of soluble IL-1α, IL-1 β and IL-1RA

Concentrations of the cytokines released by endometrial cells and ovarian cancer cells are shown in Table 2.

**Table 2:** Comparative expression of IL-1β and IL-1RA in endometrial cells and epithelial ovarian cancer cell lines

Cells	IL-1 α (pg/ml)	IL-1 β (pg/ml)	IL-1 RA (pg/ml)
**Primary EC (control)**	15.50 ± 0.4	11.00 ± 1.2	154 ± 3.9
**RL-952**	49.50 ± 2.0	13.9 ± 0.9	178 ± 4.1
**TOV-112D**	11.00 ± 0.8	22.3 ± 2.2	122 ± 2.4
**TOV-21G**	13.00 ± 1.1	21.6 ± 1.5	154 ± 1.1
**OV-90**	12.80 ± 1.4	28.3 ± 2.1	358 ± 5.3

The results of this study demonstrated the presence of IL-1α, IL-1β and IL-1RA in all cell lines. The levels of IL-1α secretion were higher in endometrial cells than ovarian cancer cells. The levels of IL-1β were significantly higher in the supernatant of EOCC than both of endometrial cells (P < 0.05). The levels of IL-1β in the supernatant of all ovarian cancer cell lines studied were significantly higher than endometrial cells (P < 0.05). Moreover, we found a high concentration of IL-1β in OV-90 cell line (P < 0.01). The levels of IL-1RA are significantly lower in EOCC compared to both endometrial cells (P < 0.05). However, IL-1RA concentrations were also lowers in EOCC compared to other ovarian cancer cell lines, with high expression in OV-90 cell line (P < 0.01).

## Discussion

Endometriosis is more often associated with ovarian cancer. The relationship with ovarian cancer can be understood as a local process of malignant transformation. It has been reported that IL-1, a pro-inflammatory cytokine, may induce immune response disorders, which thereby may contribute to the establishment and progression of ectopic endometrial implants [43,44]. Impairment of the IL-1 family cytokine network may be a cause of these immune disorders which may favor local ovarian malignant transformation in women with endometriosis.

We have measured levels of IL-1α, IL-1β and IL-1RA in endometrial and ovarian cancer cells. Our present study didn't show a significant difference expression of IL-1α cell-associated expression between ovarian endometrioid cancer cells (TOV-112D) and endometrial cells with high expression in clear cell cells (TOV-21G) (Figure 2, table 1). In contrast, IL-1α secretion levels were higher in endometrial cells than endometrioid cells (Table 2). However IL-1β was more expressed in TOV-112D cells than endometrial cells (Figure 5, table 2). These data suggested the implication of IL-1 in physiological as well as pathological processes in endometrium [9] and ovary [10,17,21].

IL-1RA which is a natural regulator of IL-1, is mainly produced by macrophages, monocytes and endometrial epithelial cells [45,46]. Previous studies have shown a deficiency of IL-1RA expression in the ectopic and eutopic endometrium of women with endometrioisis compared to healthy controls [38,47]. One of the findings of this study is the significant specific decreased levels of IL-1RA at intracellular (Figure 4; Table 1) and soluble levels (Table 2) in endometrioid ovarian cancer cell compared to endometrial and ovarian cancer cells. This is of further interest given that this subtype of ovarian cancer represents the major and the one of most commonly associated to endometriosis [2,3]. One could hypothesize that after retrograde menstruation; deficiency of IL-RA coupled to over expression of IL-1β in women with endometriosis may lead to increased stimulation of immune cells, endometrial and ectopically implanted endometrial cells. This event may accentuate the inflammatory reaction and contribute to endometrioid ovarian cancer development. Many authors reported that in peritoneal fluid, the levels of IL-8, an angiogenesis cytokine, and VEGF are increased, suggesting their role in the pathogenesis of the disease [48,49]. Furthermore, it has been shown that IL-1RA can strongly inhibit endogenous IL-8 and VEGF secretion in endometrial stromal cells [47,50]. Therefore, reduced IL-1RA levels in ectopic endometrial cells may be insufficient to inhibit the secretion of IL-8 and VEGF. These factors may facilitate their implantation and transformation to endometrioid ovarian cancer cells. IL-1β is regulated by IL-1RA and activates estrogen receptors, which increase the proliferation of breast cancer cells [51]. By this way, it is intriguing to speculate that IL-1RA deficiency coupled to IL-1beta over expression may lead to estrogen receptor over expression which is one the most markers of ovarian endometrioid subtype [52].

## Conclusions

Our findings showed that endometrioid ovarian cancer exhibited a decrease in the expression of IL-1RA, suggesting a possible link with the ectopic endometriotic tissue which has already been found deficient in expression of IL-1RA in previous studies.

## List of abbreviations

OC: ovarian cancer; EAOC: endometriosis-associated ovarian cancer; EOCC: endometrioid ovarian cancer cell.

## Competing interests

The authors declare that they have no competing interests.

## Authors' contributions

MK: A PhD student, he carried out the molecular studies and contributed in acquisition, analysis and interpretation of data and drafting the manuscript.

PB: Professor, he was involved in design, acquisition, analysis and interpretation of data.

MP: Professor, she was involved in design, acquisition, analysis and interpretation of data.

YA: Professor, he was involved in design, acquisition, analysis and interpretation of data.

AA: Professor, responsible of the project and supervisor of the research. He was involved in all steps of the work (i.e. conception, design, analysis and interpretation of data, and drafting the manuscript).

All authors read and approved the final manuscript.
